# Subarachnoid Hemorrhage Causing a Seizure: An Assessment Simulation for Medical Students

**DOI:** 10.21980/J8XH1H

**Published:** 2024-07-31

**Authors:** Robert Rainer, Blair Creedle Reynolds, Cyrus Adeli, Christopher E San Miguel

**Affiliations:** * The Ohio State University College of Medicine, Department of Emergency Medicine, Columbus, OH; ^ The Ohio State University College of Medicine, Clinical Skills Education and Assessment Center, Columbus, OH

## Abstract

**Audience:**

This simulation is intended for 4^th^ year medical students.

**Introduction:**

Headache is the fifth most common chief complaint in the emergency room, and the vast majority are ultimately diagnosed as benign primary headaches.^1^,^2^ However, subarachnoid hemorrhage (SAH) is one of several critical diagnoses which can present as a headache. With a case fatality rate of up to 66.7% in some instances, SAH is considered a “can’t miss” diagnosis.^3^

Subarachnoid hemorrhage is classically associated with a thunderclap headache, one definition of which is a headache that reaches maximal intensity within one minute or less and reaches a seven out of ten in severity.^1^ Unfortunately, a thunderclap headache is not as sensitive nor specific for SAH as is often taught. In one study, only 50% of patients with an aneurysmal subarachnoid hemorrhage presented with a thunderclap headache and an additional 19% of SAH headache came on more gradually over the course of five minutes.^4^ A second study found that only 66% of SAH patients reported a thunderclap headache.^2^ Thunderclap headaches can also be associated with other intercranial pathology including intracerebral hemorrhage, cerebral venous thrombosis, cervical artery dissection, posterior reversible encephalopathy syndrome, meningitis, and temporal arteritis among others.^1^,^2^ In a large observational study, SAH accounted for 32% of the serious pathology cases identified in patients with a thunderclap headache. Even among the thunderclap headache cohort, however, 88% of patients ultimately had a benign diagnosis (compared to 93% of patients who did not report a thunderclap headache).^2^

Additional signs and symptoms of SAH include seizures in 6–9% of patients, vomiting, neck pain and stiffness, visual disturbances, loss of consciousness, and focal cranial nerve or supratentorial deficits.^1^,^5^ A non-contrasted computer tomography (CT) of the head within six hours of headache onset can have a sensitivity of 98.7 to 100%; however, the sensitivity decreased to 86% at the 24–48 hour mark.^1^,^6^ A meta-analysis found a pooled six hour sensitivity of 1.0 and asserts that a head CT interpreted as negative by an attending radiologist effectively rules out SAH in neurologically intact patients with a defined onset of a thunderclap headache.^6^ Some guidelines in the United States still recommend shared decision making with the patient to choose between a Lumbar Puncture (LP), Computer Tomography Angiogram (CTA), or no further testing to rule out SAH in the case of a negative head CT.^2^ The more time that has elapsed between onset and CT imaging, the stronger the recommendation to pursue further testing. A negative head CT followed by a negative LP approaches 100% sensitivity for ruling out SAH, and a negative head CT with a negative CTA has a 99.4% probability of ruling out SAH.^1^,^3^ Thus it is an important learning point that if a headache has been ongoing for more than six hours and there is a high pre-test probability for an SAH, a negative head CT is not sufficient to rule out the diagnosis, and a secondary test should be ordered.

Status epilepticus is defined as five minutes of continuous seizure activity or repeated seizures without return to baseline between seizures.^7^ The immediate priorities for a seizing patient include providing supplemental oxygen, considering intubation if patient is unable to protect airway, obtaining IV access if not previously established, and checking glucose.^7^ The main priority for a patient in status epilepticus is to stop the seizure with seizure abortive medications, typically benzodiazepines, and treat life-threatening causes of status epilepticus.^7^ This simulation will enable learners to diagnose SAH that is not the classical “worst headache of my life” and manage an actively seizing patient.

**Educational Objectives:**

At the conclusion of the simulation leaners will be able to:

1. Efficiently take a history from the patient and perform a physical exam (including a complete neurological exam)
2. Identify red flag symptoms in a patient complaining of a headache
3. Order and interpret the results of a CT of the head and either a CT angiogram of the brain or a lumbar puncture to make the diagnosis of subarachnoid hemorrhage
4. Demonstrate appropriate management of a seizure
  - Check a fingerstick glucose
  - Provide supplemental oxygen
  - Administer an IV or IM benzodiazepine to treat the seizure
5. Utilize the I-PASS framework to communicate with the inpatient team during the transition of care

**Educational Methods:**

This summative simulation was designed to assess competence in two of the core Entrustable Professional Activities (EPAs), as defined by the Association of American Medical Colleges (AAMC). These include EPA 8 (Give or Receive a Patient Handover to Transition Care Responsibility) and EPA 10 (Recognize a Patient Requiring Urgent or Emergent Care and Initiate Evaluation and Management). It was performed with 4th year medical students at the conclusion of their required month-long emergency medicine (EM) clerkship. This scenario joined eight other scenarios in our pool of potential cases. These sessions are conducted using a high-fidelity manikin as the patient and a confederate/actor in the nursing role. The students complete the assessment in groups of three or four with each student acting as the team lead for one scenario. After each scenario concludes, there is a post-simulation debriefing session on the presentation, differential diagnosis, physical exam findings, and management of the target pathology. A Gather-Analyze-Summarize technique was used for the debriefing session.^8^

**Research Methods:**

Facilitators provided informal feedback to the scenario developers after the case was introduced into the assessment rotation. Learners completed a standard evaluation issued by the College of Medicine for the entire session rather than for individual scenarios. These evaluations were reviewed for the first year of implementation of this new case. Over the year, 209 students completed the summative simulation exercise, and 84 of those students completed this simulation as part of the overall exercise.

**Results:**

Overall, our facilitators felt the case fit well into our pool of simulation cases. They felt they were adequately able to assess the students’ ability to respond to a seizing patient and thought the difficulty level was appropriate for fourth year medical students. Students are asked to assess the simulation session as a whole using a standard evaluation form from the College of Medicine. The simulation assessment exercise as a whole was highly rated by the students, with 93% of students rating the overall quality of the session as Very Good or Excellent. Of the students who completed the SAH scenario, 96% rated the overall quality of the session as Very Good or Excellent. None of the comments specifically mention the SAH case.

**Discussion:**

Our department has run formative simulations during the 4th year EM clerkship for over ten years. Our primary objective is to assess 4th year students’ competence in EPA 10 (Recognize a Patient Requiring Urgent or Emergent Care and Initiate Evaluation and Management). This simulation case was written to replace another SAH case which was a more straightforward and typical presentation of a subarachnoid hemorrhage as “the worst headache of my life.” The previous case also did not require seizure management. The inclusion of the seizure management better allowed faculty to assess the students’ response to a patient’s acute decompensation, which is more in line with EPA 10, than simply making a critical diagnosis.

Our facilitators did notice that many groups initially work the patient up for meningitis but ultimately make the correct diagnosis with the lumbar puncture (LP) results. Because the students have correctly identified that the patient requires more extensive work up, and meningitis is certainly on the differential diagnoses, students are not penalized for following this line of clinical reasoning.

This simulation proved to be highly engaging for 4th year medical students, and students seemed to perform at a similar level as previous summative simulations. Overall, we felt this simulation successfully achieved the objectives of the simulation session as whole, and it was integrated into our 4th year EM clerkship simulation curriculum.

**Topics:**

Medical simulation, Emergency Medicine, Subarachnoid Hemorrhage, Intracranial Hemorrhage, Seizure, Status Epilepticus.

## USER GUIDE

[Table t1-9-3-s30]

**Table t1-9-3-s30:** 

List of Resources:	
Abstract	30
User Guide	33
Instructor Materials	36
Operator Materials	47
Debriefing and Evaluation Pearls	54
Simulation Assessment	60

**Learner Audience:**

Medical Students

**Time Required for Implementation:**

**Instructor Preparation:** 10 minutes

**Time for case:** 15 minutes

**Time for debriefing:** 10 minutes

**Recommended Number of Learners per Instructor:**

3–4 learners

**Topics:**

Medical simulation, Emergency Medicine, Subarachnoid Hemorrhage, Intracranial Hemorrhage, Seizure, Status Epilepticus.

**Objectives:**

At the conclusion of this simulation, learners will be able to:

1. Efficiently take a history from the patient and perform a physical exam (including a complete neurological exam)
2. Identify red flag symptoms in a patient complaining of a headache
3. Order and interpret the results of a CT of the head and either a CT angiogram of the brain or a lumbar puncture to make the diagnosis of subarachnoid hemorrhage
4. Demonstrate appropriate management of a seizure
  - Check a fingerstick glucose
  - Provide supplemental oxygen
  - Administer an IV or IM benzodiazepine to treat the seizure
5. Utilize the I-PASS framework to communicate with the inpatient team during the transition of care

### Linked objectives and methods

Subarachnoid hemorrhage is a life-threatening and time sensitive cause of headache requiring rapid diagnosis and appropriate management. Learners will care for a patient presenting to the Emergency Department with a chief complaint of headache and will have the opportunity to take a history and perform a physical exam (Objective 1). Learners should consider subarachnoid hemorrhage as a potential diagnosis based on the historical features of the headache, including deviation from typical migraine pattern, family history of aneurysms, and absence of a fever (Objective 2). These features should prompt the learners to order a head CT to evaluate for this etiology. When the head CT is negative, they should recognize that, due to the time course (ie, 24 hours since onset of symptoms), the patient requires either a LP or a CT angiogram of the brain to rule out the diagnosis of SAH (objective 3).The patient’s clinical status will change from her initial presentation as she has a generalized seizure, for which learners will need to initiate appropriate management, including checking a fingerstick glucose, providing supplemental oxygen, and administering an IV or IM benzodiazepine to treat the seizure. (Objective 4). Finally, learners will need to update the admitting team utilizing the I-PASS framework (Objective 5).

This scenario has been designed to assess competence in two of the core Entrustable Professional Activities (EPAs), as defined by the Association of American Medical Colleges (AAMC). These include EPA 8 (Give or Receive a Patient Handover to Transition Care Responsibility) and EPA 10 (Recognize a Patient Requiring Urgent or Emergent Care and Initiate Evaluation and Management). These objectives were tracked by facilitators utilizing an institution-specific Employee Performance evaluation form. Facilitators used this form to observe critical actions, mark performance, and take notes during the simulation for further discussion during the debriefing.

This scenario joined eight other scenarios in our pool of potential cases. At the end of each four-week clerkship, the learners are split into groups of three or four, and they complete three or four simulation scenarios as a team with each learner serving as the team leader once. These sessions are conducted using a high-fidelity manikin as the patient and a confederate/actor in the nursing role. After each scenario concludes, there is a post-simulation debriefing session on the presentation, differential diagnosis, physical exam findings, and management of the target pathology, with this case being subarachnoid hemorrhage with seizure. A Gather-Analyze-Summarize technique was used for the debriefing session.^8^

### Recommended pre-reading for instructor

The instructor should review the management of subarachnoid hemorrhage. One good resource would be “Spontaneous Subarachnoid and Intracerebral Hemorrhage,” from the 9^th^ edition of *Tintinalli’s Emergency Medicine: A Comprehensive Study Guide*.^9^ Additionally, they should review the management of status epilepticus, the “Guidelines for the evaluation and management of status epilepticus” would be a good reference.^7^ Other suggested readings include materials listed below in the “References/suggestions for further reading” section.

### Results and tips for successful implementation

This scenario was developed specifically to replace another case of Subarachnoid Hemorrhage. In the previous scenario, a patient presented to the Emergency Department with the worst headache of their life. This case was more straightforward in terms of presentation (ie, “Thunderclap Headache”) and did not require seizure management. The previous case did, however, require anticoagulation reversal. The general consensus among facilitators was that the old case did not appropriately challenge learners because the diagnosis was much clearer than in our other simulation scenarios. The previous case also did not include an acute decompensation of the patient, making it challenging to assess EPA 10. Therefore, the patient in this simulation has a history of migraines of comparable severity and does not consider the presenting headache to be the worst headache of her life. The patient also will not understand what is meant by a “thunderclap” headache if the learners use that verbiage to obtain the history. In addition, while seizure management is not explicitly listed as an expected behavior within EPA 10, our facilitators collectively agreed that the initial management of a seizure was a reasonable expectation for any medical school graduate. Therefore, we took this opportunity to add it to the case because none of other cases review this medical emergency.

Prior to using this scenario as a summative assessment, we ran a single rehearsal session. This included a group of three fourth-year medical students who volunteered to complete an additional simulation case after the completion of their summative simulation session. This group felt the case similar to the other three scenarios they had been given that day and had had equally valuable learning points. No substantial changes were made to the case after this trial run.

Since implementation in May of 2022, our facilitators feel the case fits well into our pool of simulation cases. They report being able to adequately assess the students’ ability to respond to a decompensating patient and think the difficulty level is appropriate for fourth-year medical students.

As previously described, all fourth-year medical students at our instiution complete a simulation session consisting of three to four scenarios at the conclusion of the required EM clerkship. Students are asked to assess the simulation session as a whole using a standard evaluation form from the College of Medicine. The evaluation form asks students to rate the overall quality of the session, the overall teaching quality of the instructor, and provide one or two things done well in the session and one or two things which could be done to improve the session. The first two questions are answered on a scale of Excellent-Very Good-Good-Fair-Poor. In its first year of use, 84 out of 209 (40%) students completed the subarachnoid hemorrhage scenario as part of their simulation session. The simulation session as a whole is highly rated by the students. The overall survey completion rate was 91%, and the completion rate of the SAH scenario subgroup was 90%. Of the 190 students who completed an evaluation, 93% rated the overall quality of the session as Very Good or Excellent. Of the 75 students in the SAH scenario subgroup who completed an evaluation, 96% rated the overall quality of the session as Very Good or Excellent. None of the collected comments explicitly mentioned the SAH case.

## Supplementary Information



## Figures and Tables

**Table t2-9-3-s30:** 

#1	Beta hCG Result
#2	Blood Type Result
#3	CBC Results
#4	Chem 7 Results
#5	Chem 10 Results
#6	Coagulation Studies Results
#7	Lactate Result (post-seizure)
#8	Lumbar Puncture Results
#9	Urinalysis Results
#10	Urine Drug Screen Results
#11	Venous Blood Gas Results (post-seizure)
#12	CT Head Result^10^
#13	CT Angio Brain/Neck Result^11^

**Table t3-9-3-s30:** 

**Vitals:**	Heart Rate (HR) 94	Blood Pressure (BP) 134/84
	Respiratory Rate (RR) 14	Temperature (T) 97.4°F
	Oxygen Saturation (O_2_Sat) 98% on room air	

**Table t4-9-3-s30:** 

*Beta hCG*	Negative

**Table t5-9-3-s30:** 

*Blood Type*	AB+

**Table t6-9-3-s30:** 

*Complete blood count (CBC)*	
White blood count (WBC)	10.4 × 1000/mm^3^
Hemoglobin (Hgb)	14.2 g/dL
Hematocrit (HCT)	41%
Red blood cells (RBC)	5.1
Platelet (Plt)	410 × 1000/mm^3^

**Table t7-9-3-s30:** 

*Chem 7*	
Sodium	138 mEq/L
Potassium	3.7 mEq/L
Chloride	110 mEq/L
Bicarbonate (HCO_3_)	23 mEq/L
Blood Urea Nitrogen (BUN)	13 mg/dL
Creatinine (Cr)	0.9 mg/dL
Glucose	84 mg/dL

**Table t8-9-3-s30:** 

*Chem 10*	
Calcium	9.1 mg/dL
Magnesium	2.1 mg/dL
Phosphate	3.6 mg/dL

**Table t9-9-3-s30:** 

*Coagulation Studies*	
Prothrombin time	13 seconds
Partial thromboplastin time	28 seconds
International normalized ratio	1.0

**Table t10-9-3-s30:** 

*Lactic Acid*	4.5 ml/mL

**Table t11-9-3-s30:** 

*Lumbar Puncture*	
Opening Pressure	30 mmHg
Closing Pressure	15 mmHg

**Table t12-9-3-s30:** 

Tube 1:	
White blood cells	18 WBCs/uL
RBCs in tube 1	11,153 RBCs/uL
Protein	75mg/dL
Glucose	44 mg/dL
Xanthrochromia	positive
Tube 4:	
White blood cells	16 WBCs/uL
RBCs in tube 1	10,532 RBCs/uL
Xanthrochromia	positive

**Table t13-9-3-s30:** 

*Urinalysis*	
Color	yellow
Clarity	clear
Spec gravity	1.025
pH	7.9
Protein	negative
Glucose	negative
Ketones	negative
Hemoglobin	negative
Leukocyte esterase	negative
Nitrite	negative

**Table t14-9-3-s30:** 

*Urine Toxicology Screen*	
Opiates	negative
Cocaine	negative
Marijuana	negative
PCP	negative
Amphetamines	negative
Benzodiazepines	negative
Barbiturates	negative
Methadone	negative
Propoxyphene	negative
MDMA	negative

**Table t15-9-3-s30:** 

*Venous Blood Gas (post seizure)*	
pH	7.14
pCO_2_	55 mmHg
pO_2_	63 mmHg
HCO_3_	14 mEq/L

**Figure f1-9-3-s30:**
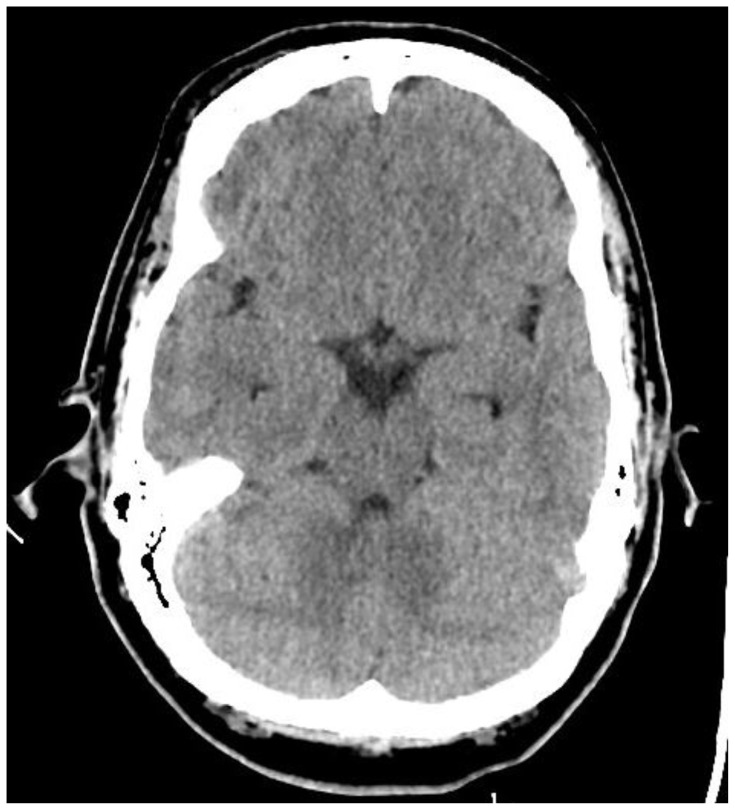
*CT IMPRESSION:* No acute intracranial hemorrhage, edema pattern or mass effect.

**Figure f2-9-3-s30:**
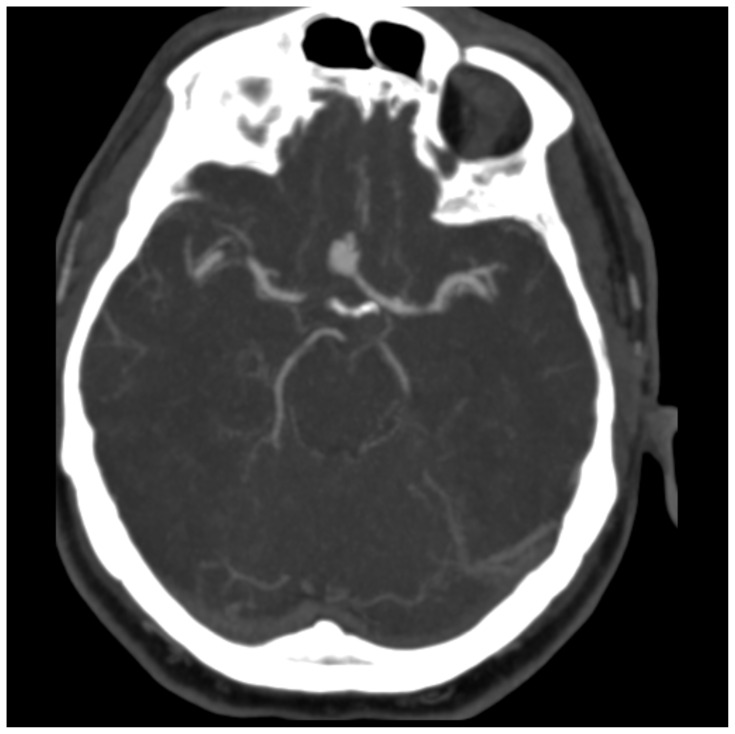
CT IMPRESSION: 15 mm aneurysm arising from the right side of the anterior communicating artery

**Table t16-9-3-s30:** SIMULATION EVENTS TABLE:

Minute (state)	Participant action/trigger	Patient status (simulator response) & operator prompts	Monitor display (vital signs)
State 1 (Baseline)	If Head CT and/or labs ordered OR 7 minutes elapsed, patient states *“I feel really strange. It’s like…”* and immediately go to State 2 (Seizure).	**Sounds/Auscultations**: Normal breath and heart sounds. **Patient Responses:** Alert and oriented x4. Speaking in full sentences, but clearly uncomfortable from the pain. The main complaint is the headache. Chief complaint: “I have a really bad headache.” Tell me more: “My migraine medication (sumatriptan) didn’t help at all, so that’s why I came in. Can you please give me something to make me feel better?” Does this feel like your typical migraine? “My migraines have been this bad before, but this feels different. Usually, my migraine is just on the right side of my head and this hurts all over.” Pain on a scale of 1 to 10: “Eight. Not the worst headache I’ve ever had.” Describe pain: “Throbbing pain. The pain became very severe in a couple of minutes and then never went away. Pain has stayed the same throughout.” What were you doing when it started? “It came out of nowhere yesterday while I was teaching my kindergarten class. It’s been steady ever since.” Would you describe it as a thunderclap headache? “I don’t know what that means.” *Only if specifically asked:* “Yes” to neck stiffness. *Only if specifically asked:* “Yes, one episode last night” to vomiting. No neuro symptoms (including weakness, numbness, vision changes, or trouble walking). No fever. **EKG on monitor**: Normal Sinus Rhythm **Appearance/PE**: Normal. Eyes wide open. Stiff neck (decreased cervical range of motion). **Learner Objectives and Interventions**: Complete history and physical exam Start treatment for headache (order medication) Order Head CT and/or labs	T 97.4° F HR 94 BP 134/84 RR 14 O2 98%
State 2 (Seizure)	If supplemental oxygen given, proceed to State 3 (Continued seizure after O2). If supplemental oxygen AND benzodiazepine given, go to State 4 (Postictal Recovery). If no supplemental oxygen given within 3 minutes in this state, go to State 5 (PEA Arrest).	**Sounds/Auscultations**: Absent bilateral breath sounds. Normal heart sounds. **Patient Responses**: Unresponsive. **EKG on monitor**: Sinus Tachycardia (with 50/60 Hz artifact). **Appearance/PE**: Seizing (clonic) with trismus enabled. Eyes closed. Not visibly breathing, no chest rise. Increasingly cyanotic. **Further details**: Confederate RN may deliver the following prompt if the learners are confused as to what they are seeing: “It looks like she’s shaking all over.” If the students ask to perform LP or send the patient for CT Angio, the nurse should say the following: “We can’t do that while she is still moving around like that.” If asked to check a POC glucose, after miming checking finger, the RN should respond, “Glucose is 79.” **Operator details**: Note: The students may give a benzo and not provide oxygen. If 3 minutes elapse without oxygen given, you should still progress to PEA Arrest, even if a benzo has been administered. **Learner Objectives and Interventions**: Ask nurse to check POC glucose Provide oxygen (nasal canula (NC), non-rebreather mask (NRB), bag valve mask (BVM), or intubation are all acceptable) Administer benzodiazepine (midazolam, lorazepam, or diazepam are all acceptable)	T 97.4° F HR 130 BP 164/96 RR 0 O2 Drift from 98% to 50% over 3 minutes (S-curve)
State 3 (Contin-ued Seizure after O2)	If benzodiazepine given, go to state 4 (Postictal Recovery). If benzodiazepine not given after 6 minutes since seizures began in State 2, go to State 6 (Comatose).	**Sounds/Auscultations**: Unchanged from State 2. **Patient Responses**: Still unresponsive. **EKG on monitor**: Unchanged from State 2. **Appearance/PE**: Unchanged from State 2. **Further details**: Facilitator may need to “fast-forward” the simulation at this point. Technically, epilepsy guidelines don’t call for treatment until 5 minutes of seizure. If the students are not actively discussing meds or intervening, the nurse can say, “The patient has now been seizing for 5 minutes.” If they were ordered, the students should receive the Head CT and any lab results in this state. Do not show LP results or CT Angio results yet. If the students ask to perform LP or send the patient for CT Angio, the nurse should say the following: “We can’t do that while she is still moving around like that.” **Operator details**: This state is identical to the previous state, except for an improved SpO_2_ level. **Learner Objectives and Interventions**: Check POC glucose (if not done already) Administer benzodiazepine (midazolam, lorazepam, diazepam are all acceptable)	T 97.4° F HR 130 BP 164/96 RR 0 O2 95%
State 4 (Postic-tal Recov-ery)	After phone call with neurosurgery, END CASE.	**Sounds/Auscultations**: Normal lung and cardiac sounds. **Patient Responses**: Patient is drowsy and confused. Unable to answer questions about her location or why she is at the hospital. “What happened?” “I want to go home.” “My head hurts.” **EKG on monitor**: Normal Sinus Rhythm (NSR) **Appearance/PE**: Eyelids half-open. Moving all extremities, but not following commands. **Further details**: If the students ordered a CT Angio study, they should receive the result in this state. If it was ordered while the patient was actively seizing, the nurse confederate can say the following: “Do you want me to order that CT Angio now?” If they have not yet ordered an LP, the nurse confederate can say the following: “What do you think caused her to have a seizure?” If the students ask for a neurology consult, the facilitator should call in, hear the story, and say the following: “It will be a minute before I can be there -- I’m busy with stroke alerts. Go ahead and load her with a gram of levetiracetam. What do you think is her underlying problem?” **Learner Objectives and Interventions**: Communicate the diagnosis of SAH in the handoff Consult neurosurgery and admit patient to the proper service (ICU, OR, or endovascular suite)	T 97.4° F HR 96 BP 130/44 RR 14 O2 95%
State 5 (PEA)	After completion of 1 round of ACLS while providing supplemental O2, go to State 6 (Comatose). If completion of 3 rounds of ACLS without providing supplemental O2, END CASE.	**Sounds/Auscultations**: Absent **Patient Responses**: Unresponsive **EKG on monitor**: Sinus Bradycardia **Appearance/PE**: Eyes closed. **Operator details:** If learners perform adequate compressions, click *“Good Compressions”* handler (sets EtCO2 to 22 and SpO2 to 92%). **Further details**: After two rounds of Advanced cardiac life support (ACLS), if the students are not bagging or have not placed an advanced airway, the nurse confederate should say the following: “What do you think caused her to arrest?” As with other simulation cases, the facilitator may need to fast-forward time between pulse checks. At the facilitator’s discretion, if the students are not actively discussing the case or intervening, the nurse confederate can say the following: “It’s been two minutes since the last pulse check.” **Learner Objectives and Interventions**: Perform high quality ACLS with minimal interruptions in chest compression, an early pulse check, and promptly address the patient’s hypoxia (ie, within 2 rounds of ACLS). Patient will improve with BVM, laryngeal mask airway (LMA) or intubation.	T 97.4° F HR 25 BP 0/0 RR 0 O2 0 EtCO2 12
State 6 (Comatose)	After phone call with neurosurgery, END CASE.	**Sounds/Auscultations**: Normal lung and cardiac sounds. **Patient Responses**: Unresponsive If learners ask to check a gag reflex, the sim tech should verbalize a dry-heaving sound. **EKG on monitor**: NSR **Appearance/PE**: Eyes closed. Patient withdraws to pain in all four extremities. **Further details**: If the students ordered a CT Angio study, they should receive the result in this state. If it was ordered while the patient was actively seizing, the nurse confederate can say the following: “Do you want me to order that CT Angio now?” If they have not yet ordered an LP, the nurse confederate can say the following: “What do you think caused her to have a seizure?” If the students ask for a neurology consult, the facilitator should call in, hear the story, and say the following: “I’m busy with stroke alerts, so it will be a minute before I can be there. Go ahead and load her with a gram of levetiracetam. What do you think is her underlying problem?” **Learner Objectives and Interventions:** Communicate the diagnosis of SAH in the handoff Consult neurosurgery and admit patient to the proper service (ICU, OR, or endovascular suite)	T 97.4° F HR 115 BP 156/84 RR 26 O2 98% EtCO2 32

**Figure:**
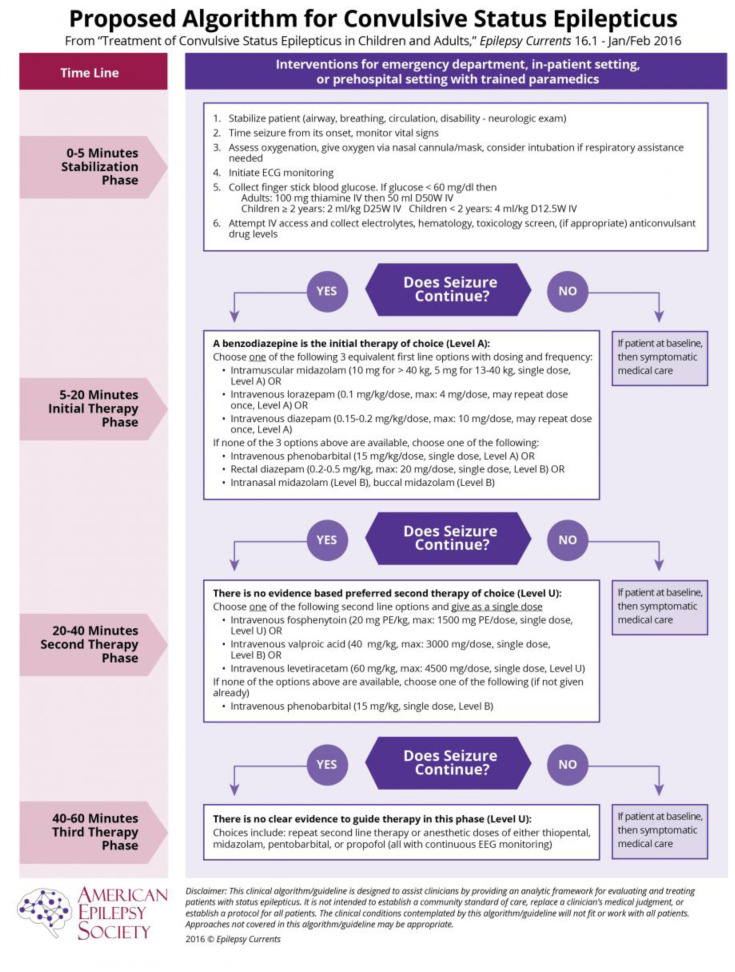


**Table t17-9-3-s30:** Exemplar I-PASS Sign-out For Leticia Mendoza-Ruiz

**I**	Illness Severity	Stable and/or Postictal
**P**	Patient Summary	This patient presented with 1 day of a thunderclap headache. Her non-contrasted head CT was normal, but the CT Angiogram showed a 15 mm aneurysm of her anterior communicating artery, so I believe she may have a subarachnoid hemorrhage. She then had a generalized seizure that lasted 3 minutes and aborted with 4 mg of Lorazepam IV. While her mental status is improving, she is not yet back to her neurological baseline.
**A**	Action List	Patient is going to need monitoring for seizures, blood pressure control, and ultimately definitive management of her aneurysm.
**S**	Situation Awareness and Contingency Planning	If patient has another seizure or does not regain her mental status within 2 hours, I would consider rescanning her head to evaluate for rebleed.
**S**	Synthesis by Receiver	From the Facilitator: “So this patient came in with a thunderclap headache, presumably from a subarachnoid hemorrhage, had a seizure, and is now postictal. We will need to monitor her mental status and take her either to the OR or endovascular suite to treat the aneurysm.”

**Table t18-9-3-s30:** 

**Critical Actions:** Perform a focused history and physical exam (including obtaining vital signs) Consider subarachnoid hemorrhage as a potential diagnosis Order CT Head Perform LP or obtain CTA Brain Check fingerstick glucose Provide supplemental oxygen Consult neurosurgery and admit patient to proper service (ICU, OR, or endovascular suite)	0:00

**Table t19-9-3-s30:** 

	Milestone	Did not achieve level 1	Level 1	Level 2	Level 3
1	**Emergency Stabilization (PC1)**	□ Did not achieve Level 1	□ Recognizes abnormal vital signs	□ Recognizes an unstable patient, requiring intervention Performs primary assessment Discerns data to formulate a diagnostic impression/plan	□ Manages and prioritizes critical actions in a critically ill patient Reassesses after implementing a stabilizing intervention
2	**Performance of focused history and physical (PC2)**	□ Did not achieve Level 1	□ Performs a reliable, comprehensive history and physical exam	□ Performs and communicates a focused history and physical exam based on chief complaint and urgent issues	□ Prioritizes essential components of history and physical exam given dynamic circumstances
3	**Diagnostic studies (PC3)**	□ Did not achieve Level 1	□ Determines the necessity of diagnostic studies	□ Orders appropriate diagnostic studies. Performs appropriate bedside diagnostic studies/procedures	□ Prioritizes essential testing Interprets results of diagnostic studies Reviews risks, benefits, contraindications, and alternatives to a diagnostic study or procedure
4	**Diagnosis (PC4)**	□ Did not achieve Level 1	□ Considers a list of potential diagnoses	□ Considers an appropriate list of potential diagnosis May or may not make correct diagnosis	□ Makes the appropriate diagnosis Considers other potential diagnoses, avoiding premature closure
5	**Pharmacotherapy (PC5)**	□ Did not achieve Level 1	□ Asks patient for drug allergies	□ Selects an medication for therapeutic intervention, consider potential adverse effects	□ Selects the most appropriate medication and understands mechanism of action, effect, and potential side effects Considers and recognizes drug-drug interactions
6	**Observation and reassessment (PC6)**	□ Did not achieve Level 1	□ Reevaluates patient at least one time during case	□ Reevaluates patient after most therapeutic interventions	□ Consistently evaluates the effectiveness of therapies at appropriate intervals
7	**Disposition (PC7)**	□ Did not achieve Level 1	□ Appropriately selects whether to admit or discharge the patient	□ Appropriately selects whether to admit or discharge Involves the expertise of some of the appropriate specialists	□ Educates the patient appropriately about their disposition Assigns patient to an appropriate level of care (ICU/Tele/Floor) Involves expertise of all appropriate specialists
9	**General Approach to Procedures (PC9)**	□ Did not achieve Level 1	□ Identifies pertinent anatomy and physiology for a procedure Uses appropriate Universal Precautions	□ Obtains informed consent Knows indications, contraindications, anatomic landmarks, equipment, anesthetic and procedural technique, and potential complications for common ED procedures	□ Determines a back-up strategy if initial attempts are unsuccessful Correctly interprets results of diagnostic procedure
20	**Professional Values (PROF1)**	□ Did not achieve Level 1	□ Demonstrates caring, honest behavior	□ Exhibits compassion, respect, sensitivity and responsiveness	□ Develops alternative care plans when patients’ personal beliefs and decisions preclude standard care
22	**Patient centered communication (ICS1)**	□ Did not achieve level 1	□ Establishes rapport and demonstrates empathy to patient (and family) Listens effectively	□ Elicits patient’s reason for seeking health care	□ Manages patient expectations in a manner that minimizes potential for stress, conflict, and misunderstanding. Effectively communicates with vulnerable populations, (at risk patients and families)
23	**Team management (ICS2)**	□ Did not achieve level 1	□ Recognizes other members of the patient care team during case (nurse, techs)	□ Communicates pertinent information to other healthcare colleagues	□ Communicates a clear, succinct, and appropriate handoff with specialists and other colleagues Communicates effectively with ancillary staff

## INSTRUCTOR MATERIALS

**Case Title:** Subarachnoid Hemorrhage with Seizure

**Case Description & Diagnosis (short synopsis):** The scenario begins with the patient presenting to the Emergency Department with a chief complaint of headache. Although the pain will be severe, the patient will be able to have a conversation with the learners and fully participate in a physical exam. After the learners have ordered their tests but before any results are available, the patient will have a generalized seizure. The learners will need to stabilize the patient in order to complete the work-up and make the definitive diagnosis. If not already decided based on the history alone, the seizure should prompt the learners to perform a more extensive work-up for this particular headache. In order to make the correct diagnosis, the learners will need to order either a CT angio study or a lumbar puncture. Ultimately the patient will be admitted to neurosurgery for operative management.

**Equipment or Props Needed:**

- High fidelity simulator (this study used a SimMan® 3G Manikin)
- Hospital-grade defibrillator
- Cardiac monitor
- Pulse oximetry
- IV pole
- Peripheral IV line
- Crash cart with ambu-bag and intubation kit
- Non-rebreather
- Nasal cannula
- Syringe
- Glucometer
- Three 1 L bags of fluid (either NS or LR)
- Other simulated medications with labeling (not limited to: Lorazepam (Ativan) syringe, Ketorolac (Toradol) syringe, Levetiracetam (Keppra) bag, Midazolam (Versed) syringe, Ceftriaxone (Rocephin) bag, Vancomycin bag, Cefepime bag, Piperacillin/tazobactam (Zosyn) bag, Diazepam (Valium) syringe, Succinylcholine syringe, Etomidate syringe, Ketamine syringe, Rocuronium syringe, Epinephrine syringe, Ondansetron (Zofran) tablet or syringe, Prochlorperazine (Compazine) syringe, Diphenhydramine (Benadryl) tablet or syringe)

**Actors needed:**

One actor in the nursing role. The facilitator will act as the consultant via the overhead speaker: Neurosurgery, Neurology, and/or Pharmacy. The three to four learners will act as Emergency Medicine attendings, with one learner acting as the team leader.

**Stimulus Inventory:** [Table t2-9-3-s30]

**Background and brief information:** The patient is a 28-year-old female presenting to the Emergency Department with a headache. The three to four learners will act as Emergency Medicine attendings, with one learner acting as the team leader.

**Initial presentation:** The patient is overall well-appearing. She is alert and oriented, and speaking in full sentences, but clearly uncomfortable from the pain. She is complaining of headache. The onset of symptoms was acute (over a few minutes) and occurred yesterday while she was teaching her kindergarten class. The headache persisted this morning, which prompted her to come to the Emergency Department. She describes the pain as throbbing and rates the pain as 8/10 in severity, but she has had headaches of a similar pain level previously. However, she states that this headache was unique because it happened so quickly, and it involved her entire head. Usually, her migraines only affect the right side of her head. She took her sumatriptan without relief. There are no aggravating or alleviating factors. Upon questioning, she endorses neck stiffness and one episode of non-bloody, non-bilious emesis last night. She denies trauma, fevers, visual changes, weakness, numbness, or difficulty walking.

**How the scene unfolds:** After the learners place their initial diagnostic and therapeutic orders, the patient will suffer a seizure. The learners will need to stabilize the patient in order to complete the work-up and make the definitive diagnosis. The learners will need to provide supplemental oxygen, obtain a fingerstick glucose, and administer benzodiazepine to stabilize the patient. Without oxygen, the patient will suffer a hypoxic cardiac arrest. Without benzodiazepine, the patient will become comatose.

Once the patient is stabilized, they can turn their attention again to the underlying diagnosis. The red flag features in this case include deviation from her typical migraine pattern, a family history of aneurysms, and eventually the presence of a seizure. These features should prompt the learners to order a head CT to evaluate for this etiology. When the head CT is negative, they should recognize that, due to the time course (ie, 24 hours since onset of symptoms), the patient requires either a lumbar puncture or a CT angiogram of the brain to rule out the diagnosis of SAH. Once the diagnosis of subarachnoid hemorrhage is made, learners should consult neurosurgery and communicate the diagnosis during handoff. The patient should be admitted to the neurosurgery service.

**Critical actions:**

1. Perform a focused history and physical exam (including obtaining vital signs)
2. Consider subarachnoid hemorrhage as a potential diagnosis
3. Order CT Head
4. Perform LP or obtain CTA Brain
5. Check fingerstick glucose
6. Provide supplemental oxygen
7. Consult neurosurgery and admit patient to proper service (ICU, OR, or endovascular suite)

**Case Title:** Subarachnoid Hemorrhage with Seizure

**Chief Complaint:** Leticia Mendoza-Ruiz is a 28-year-old female who is presenting with a headache.[Table t3-9-3-s30]

**General Appearance:** Alert and interactive, but clearly uncomfortable from the pain.

**Primary Survey:**

- **Airway:** Speaking in full sentences
- **Breathing:** Lungs clear to auscultation bilaterally
- **Circulation:** 2+ pulses to all extremities

**History:**

- **History of present illness:** 28-year-old female who presents with a headache. Patient states that she first noticed the headache yesterday morning. The headache came out of nowhere while she was teaching her kindergarten class about the colors of the rainbow. When she woke up this morning, the headache was still there, which prompted her to come to the Emergency Department. She describes the pain as throbbing and rates the pain as 8/10 in severity, but she has had headaches of a similar pain level previously. However, she states that this headache was unique because it happened so quickly, and it involved her entire head. Usually, her migraines only affect the right side of her head. She took her sumatriptan without relief. There are no aggravating or alleviating factors. Upon questioning, she endorses neck stiffness and one episode of non-bloody, nonbilious emesis last night. She denies trauma, fevers, visual changes, weakness, numbness, or difficulty walking.
- **Past medical history:** Migraines (age 15), Asthma (age 3), Seasonal allergies (age 12)
- **Past surgical history:** Appendectomy (age 24)
- **Patient’s medications:** Sumatriptan (Imitrex) for migraines, Cetirizine (Zyrtec) for seasonal allergies
- **Allergies:** Strawberries
- **Social history:** None
- **Family history:** None

**Secondary Survey/Physical Examination:**

- **General appearance:** Overall well-appearing, but clearly uncomfortable from the pain.
- **HEENT:** Within Normal Limits (WNL)
- **Neck:** Neck Stiffness
- **Heart:** WNL
- **Lungs:** WNL
- **Abdominal/GI:** WNL
- **Genitourinary:** WNL
- **Rectal:** WNL
- **Extremities:** WNL
- **Back:** WNL
- **Neuro:** WNL
- **Skin:** WNL
- **Lymph:** WNL
- **Psych:** WNL

[Table t4-9-3-s30] [Table t5-9-3-s30] [Table t6-9-3-s30] [Table t7-9-3-s30] [Table t8-9-3-s30] [Table t9-9-3-s30] [Table t10-9-3-s30] [Table t11-9-3-s30] [Table t12-9-3-s30] [Table t13-9-3-s30] [Table t14-9-3-s30] [Table t15-9-3-s30]

*CT Head (non-contrast)*

Glick Y, Normal CT head. Case study, Radiopaedia.org (Accessed on 21 Jul 2024)

https://doi.org/10.53347/rID-178062 [Fig f1-9-3-s30]

*CT Angio Head*

Di Muzio B, Ruptured anterior communicating artery aneurysm. Case study, Radiopaedia.org (Accessed on 21 Jul 2024) https://doi.org/10.53347/rID-37775[Fig f2-9-3-s30]

## OPERATOR MATERIALS

[Table t16-9-3-s30]

**Diagnosis:**

Subarachnoid Hemorrhage

**Disposition:**

Admission to Neurosurgery (ICU, OR, or endovascular suite)

## DEBRIEFING AND EVALUATION PEARLS

### Subarachnoid Hemorrhage Causing a Seizure

**Pearls:**

1. **Efficiently take a history from the patient and perform a physical exam**
  - When evaluating a patient with a headache, there are several key history and physical exam elements that the student should focus on. These include the time course, severity, and location of the pain; a personal history of headaches, or clotting disorders; whether the patient takes blood thinning medication; a family or personal history of aneurysms; associated symptoms of neck stiffness, fever, vision changes, and neurological deficits.
  - The student should perform HEENT and full neurological exams.
2. **Identify subarachnoid hemorrhage (SAH) as a potential diagnosis**
  - Subarachnoid hemorrhage is a “can’t miss” diagnosis that often presents with a headache. Classically, SAH is associated with a “thunderclap” headache, meaning the headache is very painful and peaks in severity within a minute of onset. Not all patients with SAH, however, report an onset quite this quickly, but 100% of patients in one case-series reported maximal onset within the first hour.^12^
  - Stiff neck, family or personal hx of aneurysms, and onset with physical exertion are other features that should make the clinician concerned for SAH.
  - A normal neurological exam can be falsely reassuring, as up to 50% of patients with SAH have normal neurological exams.^13^
  - Management of SAH
    - Beyond simply consulting neurosurgery, management of SAH is not an emphasis of this simulation because it is beyond the scope of most practicing physicians. However, a quick review of SAH management may be appropriate for groups of learners who have mastered basic treatment concepts.
    - Blood pressure control
      1. This is a balancing act between reducing the blood pressure (to reduce the risk of rebleeding), while at the same time ensuring that you are maintaining an adequate cerebral perfusion pressure (MAP-ICP).
      2. For most patients, the BP goals should be < 160 mmHg systolic or a MAP < 110 mmHg.
    - Reverse coagulopathy
      1. If the patient is on blood thinners, they should be given the appropriate reversal agents, if available.
      2. If thrombocytopenic, the patient should receive platelets to a level of 50–100K. Platelet transfusion in the setting of antiplatelet agents is a bit controversial, due to the inflammation cascade that is often triggered by platelet transfusion.
    - Ultimately, the patient will require stabilization of the aneurysm using either an open approach (ie, clipping), or an endovascular approach (ie, coiling). Both of these procedures are primarily performed by neurosurgery services.
3. **Order a CT of the head and either a CT angiogram of the brain or a lumbar puncture to make the diagnosis**
  - Classically, the work up for this pathology has been a Head CT followed by a lumbar puncture if the head CT was normal.
    - As CT technology has advanced, there are now several studies which demonstrate that a head CT within 6 hours of headache onset is nearly 100% sensitive for SAH.^6^ This sensitivity, however, decreases with time because the appearance of the blood loses its natural contrast from surrounding tissue.
    - Therefore, if there is a high suspicion for SAH, another test is required to rule it out.
  - A lumbar puncture in the setting of SAH should have an elevated opening pressure, a red blood cell count that does not diminish between tubes 1 and 4, and xanthochromia.
    - No diagnostic cut-offs for red blood cells have been established, but less than 100 RBC/μL is unlikely to be caused by SAH.
    - Xanthochromia is a pink or yellow tint caused by hemoglobin degradation products. This can be seen as early as two hours after the onset of SAH. By hour 12, 100% of SAH patients have grossly visible xanthochromia in the CSF.^14^
  - Given the risk of ambiguous results after a lumbar puncture (or no results at all if the procedure is unsuccessful), some clinicians advocate for performing CT Angio study in place of the lumbar puncture. Rather than looking for SAH, they are instead looking for cerebral aneurysms, whose rupture would have caused the SAH.
  - The downside to this approach is that the clinician may find aneurysms that weren’t actually bleeding and otherwise wouldn’t need intervention. Also, while the test characteristics for cerebral aneurysm are very good, it is not quite as good as the gold standard, diagnostic cerebral angiogram.
  - This approach is unlikely to diagnose non-aneurysmal SAH.
4. **Demonstrate appropriate management of a seizure**
  - Check a fingerstick glucose
    - Although this patient has a clear cause of her seizure, checking a POC glucose should be part of the ingrained process of managing an acute seizure.
    - Treatment for hypoglycemia is generally recommended if the level is < 60 mg/dl.
    - For adults with an IV, give 1 amp (25 grams) of D50%. For adults without an IV, give 1 mg of Glucagon IM.
    - Pediatric dosing is a bit more complicated and in an emergent setting, providers should follow the Broselow tape dosing.
      1. Infants and children < 5 years: 2.5 to 5 mL/kg of 10% dextrose solution (D10W)
      2. Children greater than 5 years of age: 1 to 2 mL/kg of 25% dextrose (D25W)
    - Of note, even in known alcoholics, you should not delay the administration of glucose in order to give thiamine first. Requiring thiamine prior to glucose administration is an outdated dogma^15^
  - Provide supplemental oxygen
    - An active, generalized seizure inhibits normal muscle contraction for breathing. Supplemental oxygen can be used to help avoid hypoxia.
    - Usually, a non-rebreather is the best choice because the nasal cannula is easily dislodged and does not provide as much oxygen.
    - In prolonged seizures, providers may need to assist respirations via bagging. They should also prepare suction and be prepared to turn the patient on their side in case the patient begins vomiting. Most emergency physicians will consider intubating the patient once they have proceeded beyond the benzo dosing in the below algorithm.^16^
  - Administer an IV or IM benzodiazepine to treat the seizure
    - There are several acceptable treatment regimens. Most commonly, lorazepam (brand name Ativan) is used at 0.1 mg/kg/dose with a max dose of 4mg. This can be repeated once.
    - If the seizure continues, the physician should give IV fosphenytoin, IV valproic acid, or IV levetiracetam (brand name Keppra).
    - Use the below algorithm to guide treatment of status epilepticus.^16^
5. **Utilize the I-PASS framework to communicate with the inpatient team during the transition of care**
  - Illness Severity – Describe whether the patient is stable, unstable, or someone that may decompensate
  - Patient Summary – Give a summary statement and explain the hospital course up until now
  - Action List – Explain what still needs to be done for the patient
  - Situation Awareness And Contingency Plans – Highlight any potential changes that may occur in the patient’s clinical status and what could be done if they were to happen
  - Synthesis by Receiver- Utilizing closed loop communication, the receiver explains their understanding of the situation to the one giving sign-out
  - When reflecting on the sign-out given at the beginning of this case, one can see how unhelpful it was for understanding what is happening with the patient. When looking at the example below, you can understand why this framework is a much more effective form of communication.[Table t17-9-3-s30]

## SIMULATION ASSESSMENT

*Subarachnoid Hemorrhage with Seizure*

Learner: _________________________________________

***Assessment Timeline***

This timeline is to help observers assess their learners. It allows observer to make notes on when learners performed various tasks, which can help guide debriefing discussion.[Table t18-9-3-s30]

**Critical Actions:**

□ Perform a focused history and physical exam (including obtaining vital signs)
□ Consider subarachnoid hemorrhage as a potential diagnosis
□ Order CT Head
□ Perform LP or obtain CTA Brain
□ Check fingerstick glucose
□ Provide supplemental oxygen
□ Consult neurosurgery and admit patient to proper service (ICU, OR, or endovascular suite)

**Summative and formative comments:**

**Milestones assessment:** [Table t19-9-3-s30]
