# Supplementary material for: Subarachnoid Hemorrhage Causing a Seizure: An Assessment Simulation for Medical Students
Source: J Educ Teach Emerg Med. 2024 Jul 31;9(3):S30–64. doi: 10.21980/J8XH1H (PMC11312874; doi:10.21980/J8XH1H)
Supplement: Supplementary file 1 [file 9-3-S30-Supp1.pptx]

## Slide 1
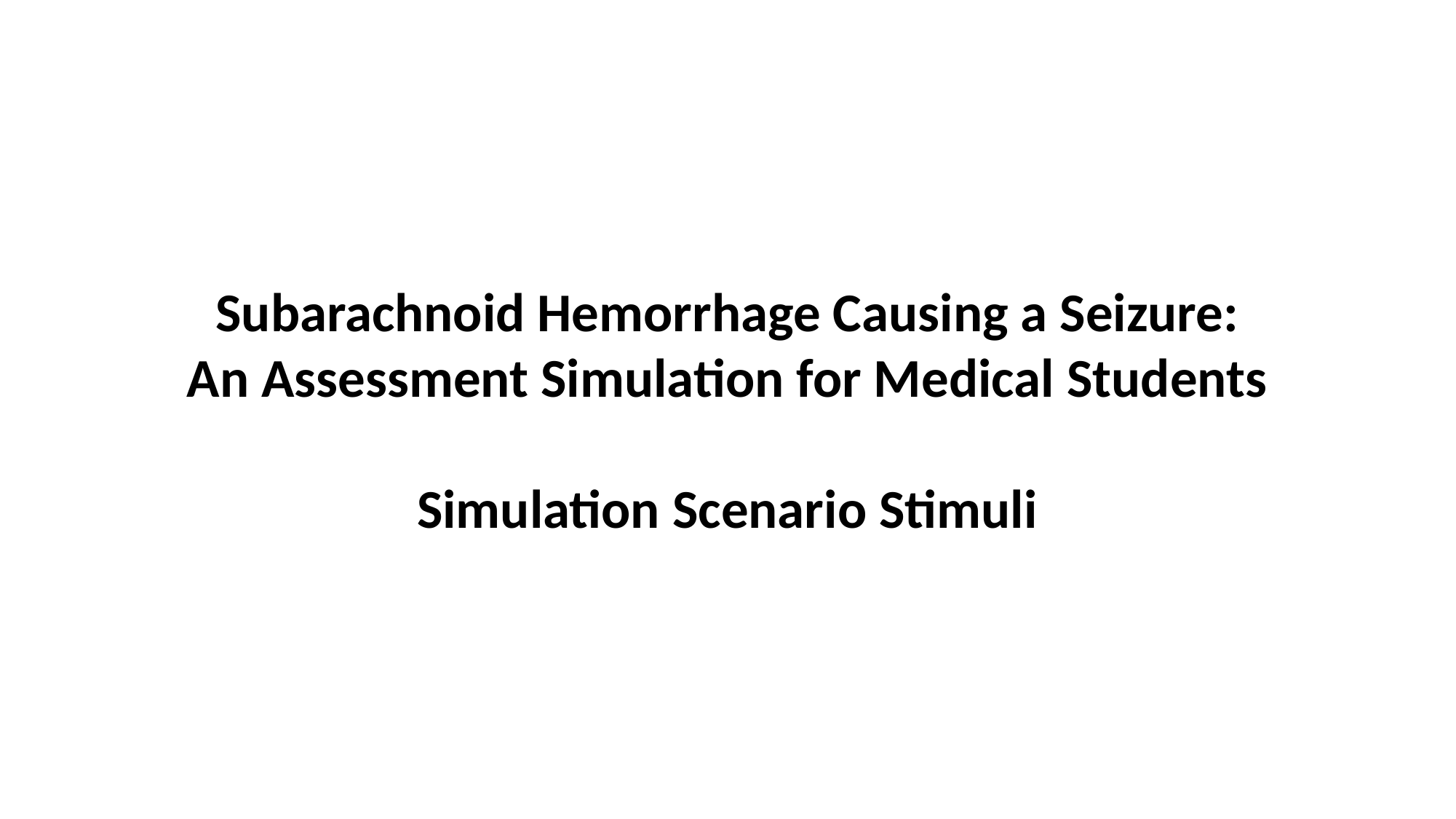

Subarachnoid Hemorrhage Causing a Seizure:
An Assessment Simulation for Medical Students
Simulation Scenario Stimuli

## Slide 2
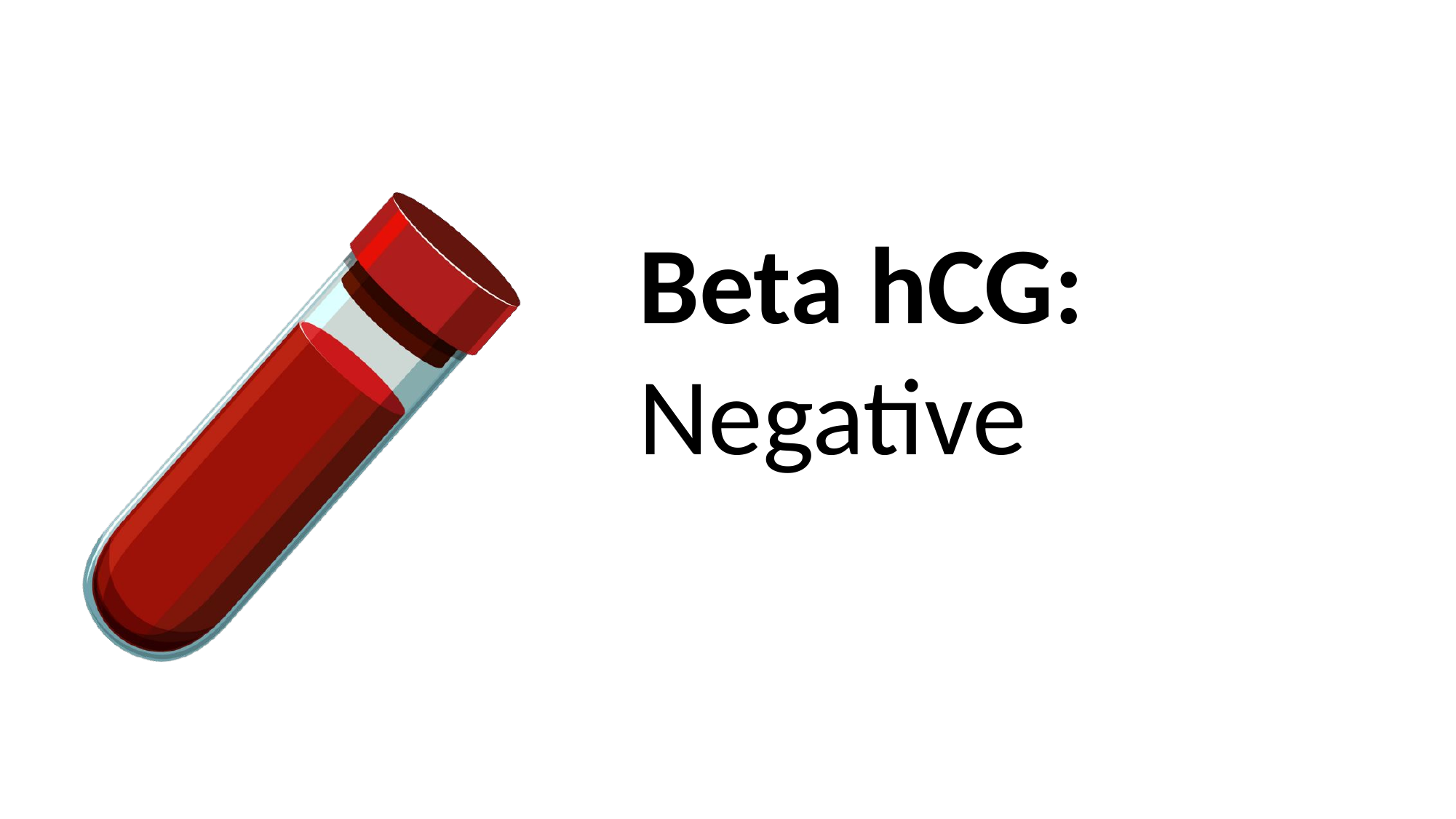

Beta hCG:
Negative

## Slide 3
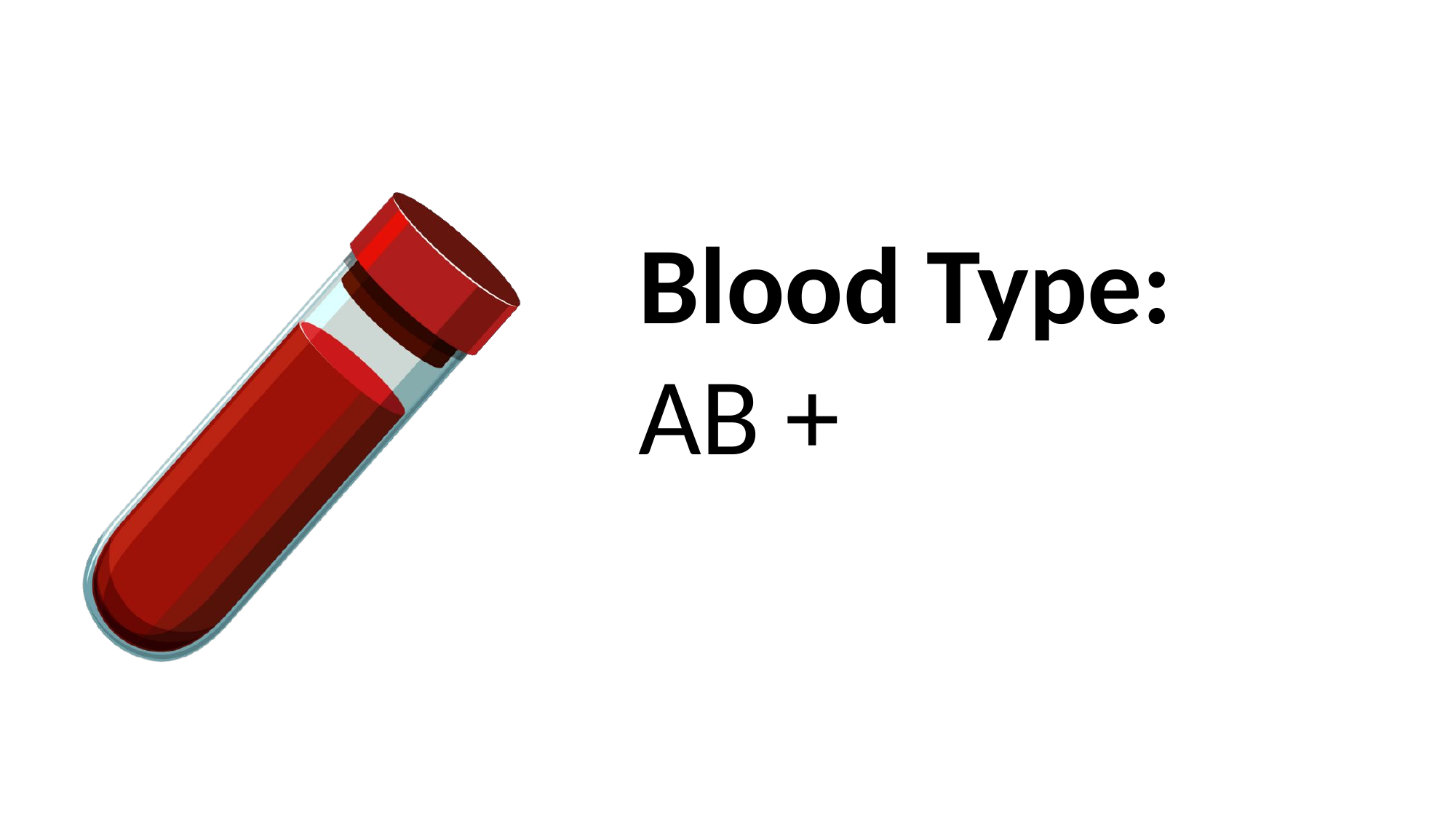

Blood Type:
AB +

## Slide 4
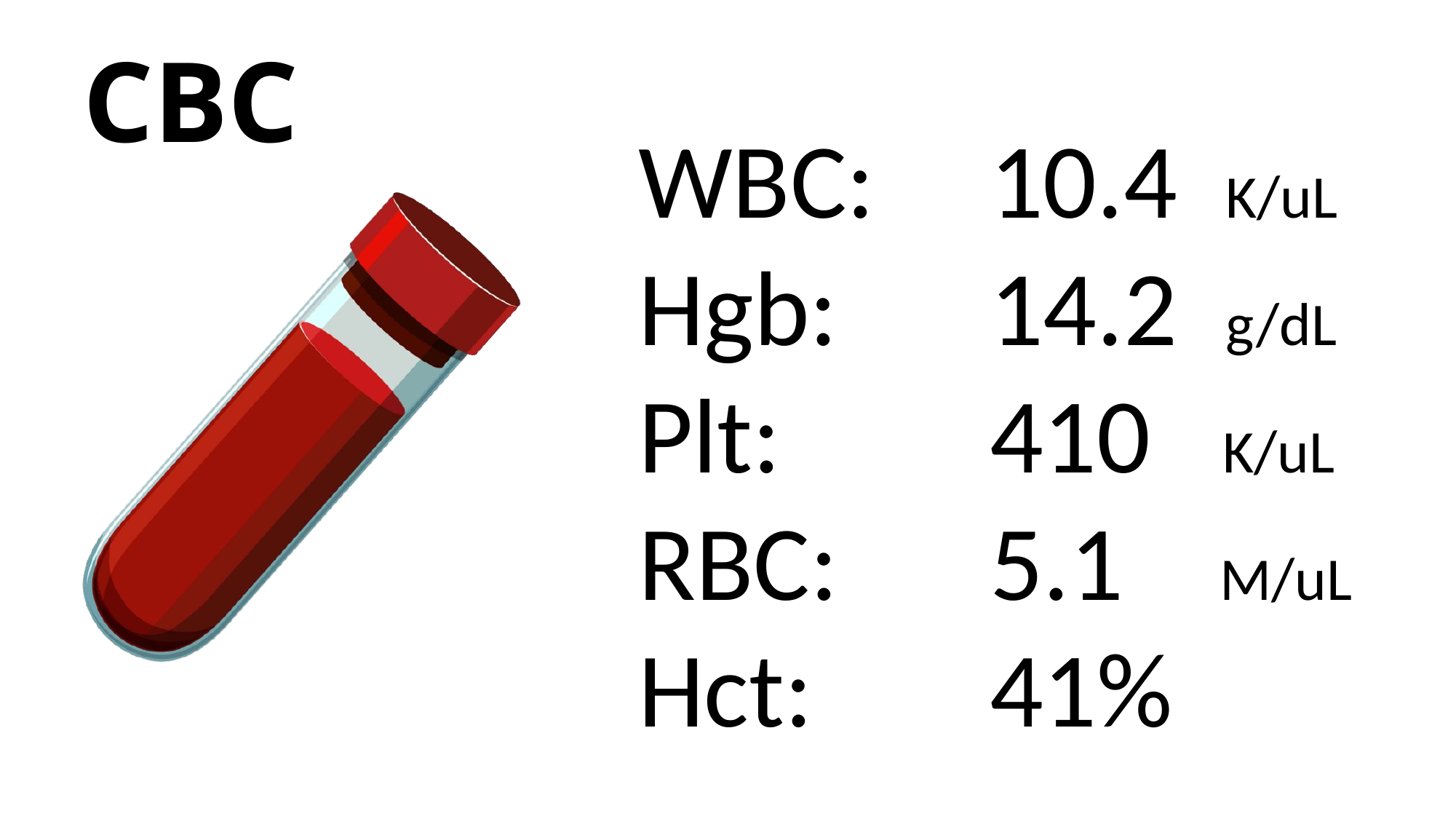

# CBC
WBC:	 10.4 K/uL
Hgb:	 	 14.2 g/dL
Plt:		 410 K/uL
RBC:		 5.1 M/uL
Hct:		 41%

## Slide 5
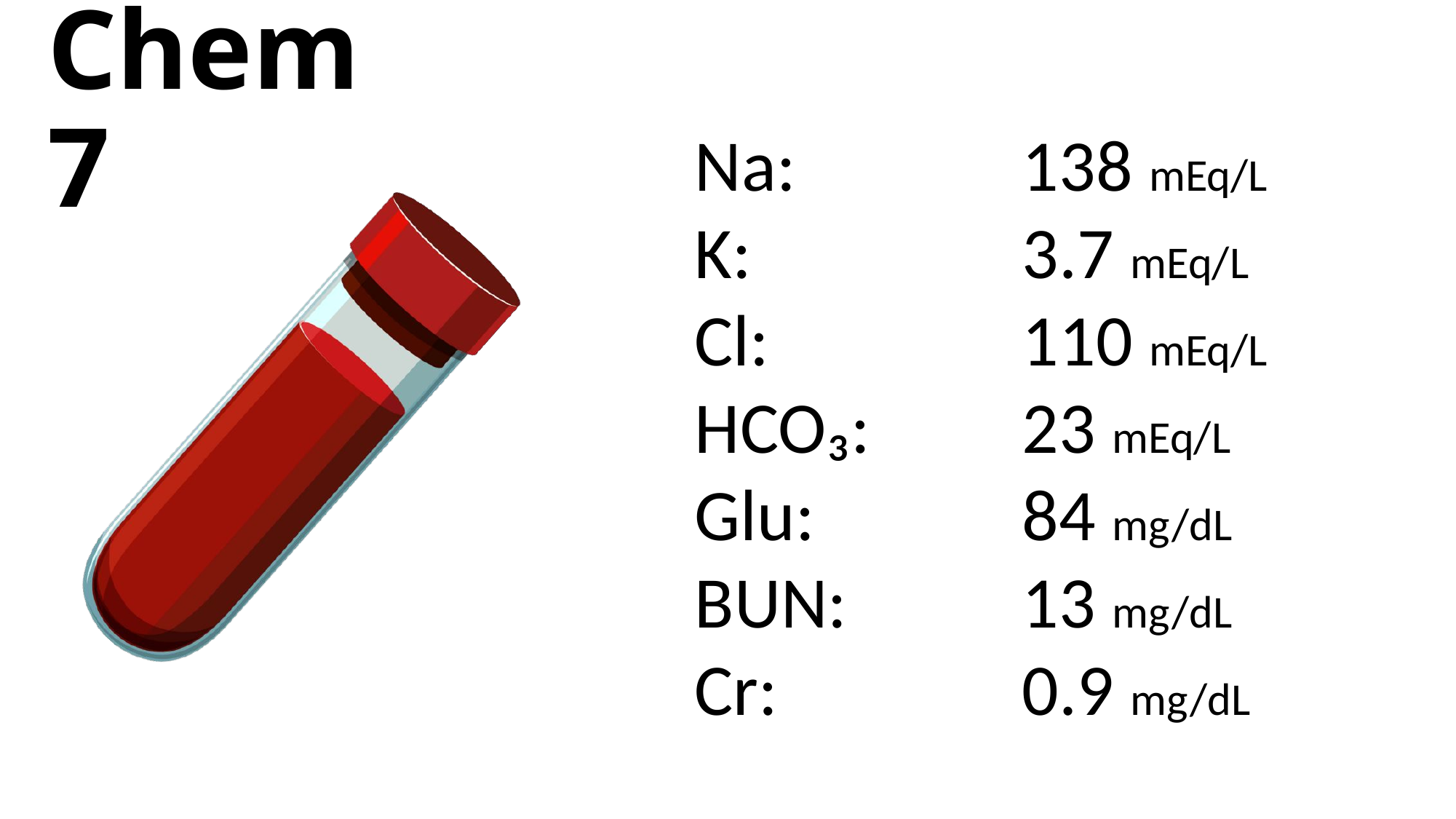

# Chem 7
Na:	 		138 mEq/L
K:			3.7 mEq/L
Cl:			110 mEq/L
HCO₃:		23 mEq/L
Glu:		84 mg/dL
BUN: 		13 mg/dL
Cr: 	 		0.9 mg/dL

## Slide 6
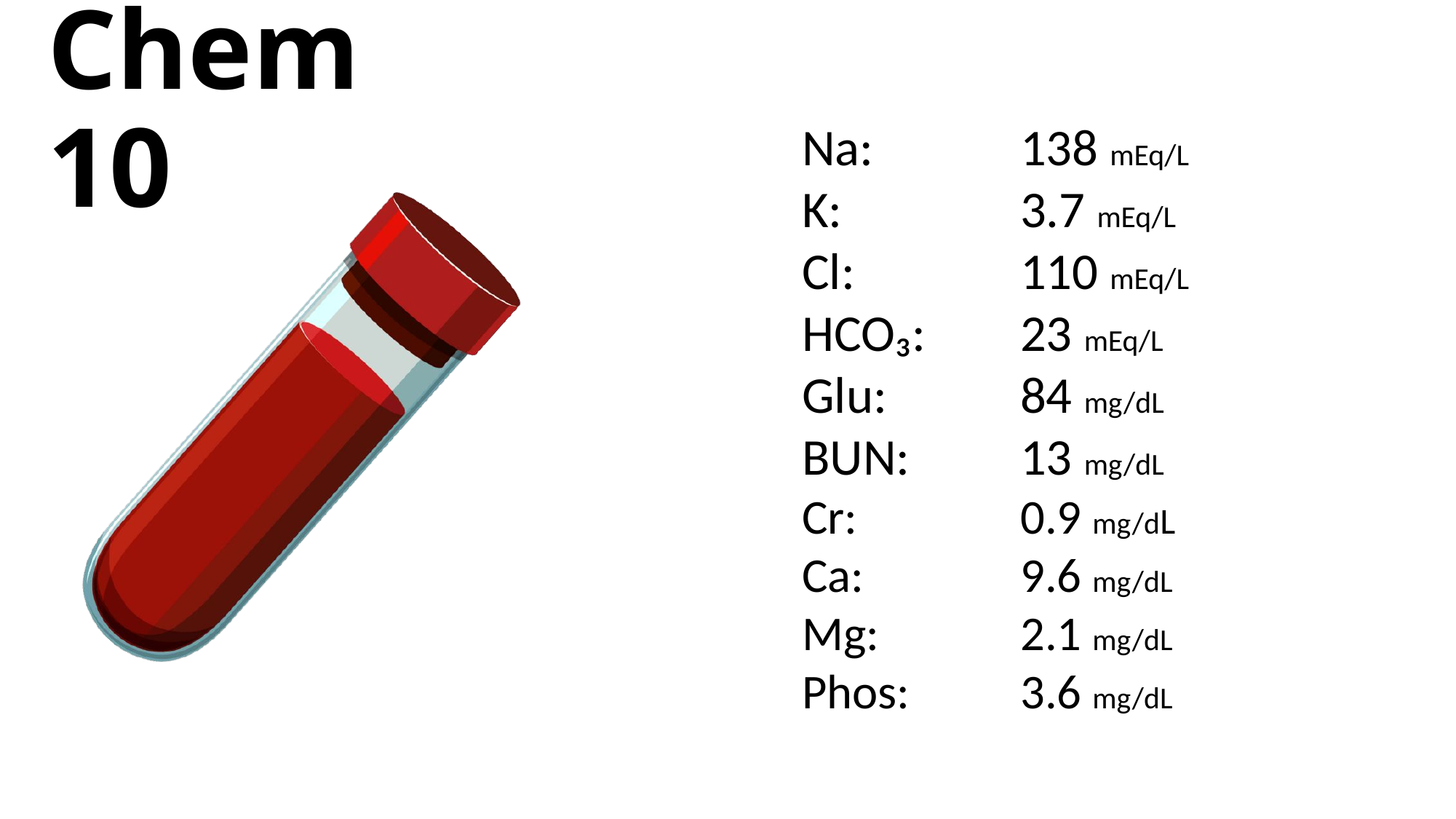

# Chem 10
Na:	 	138 mEq/L
K:		3.7 mEq/L
Cl:		110 mEq/L
HCO₃:	23 mEq/L
Glu:		84 mg/dL
BUN: 	13 mg/dL
Cr: 	 	0.9 mg/dL
Ca:		9.6 mg/dL
Mg:		2.1 mg/dL
Phos:		3.6 mg/dL

## Slide 7
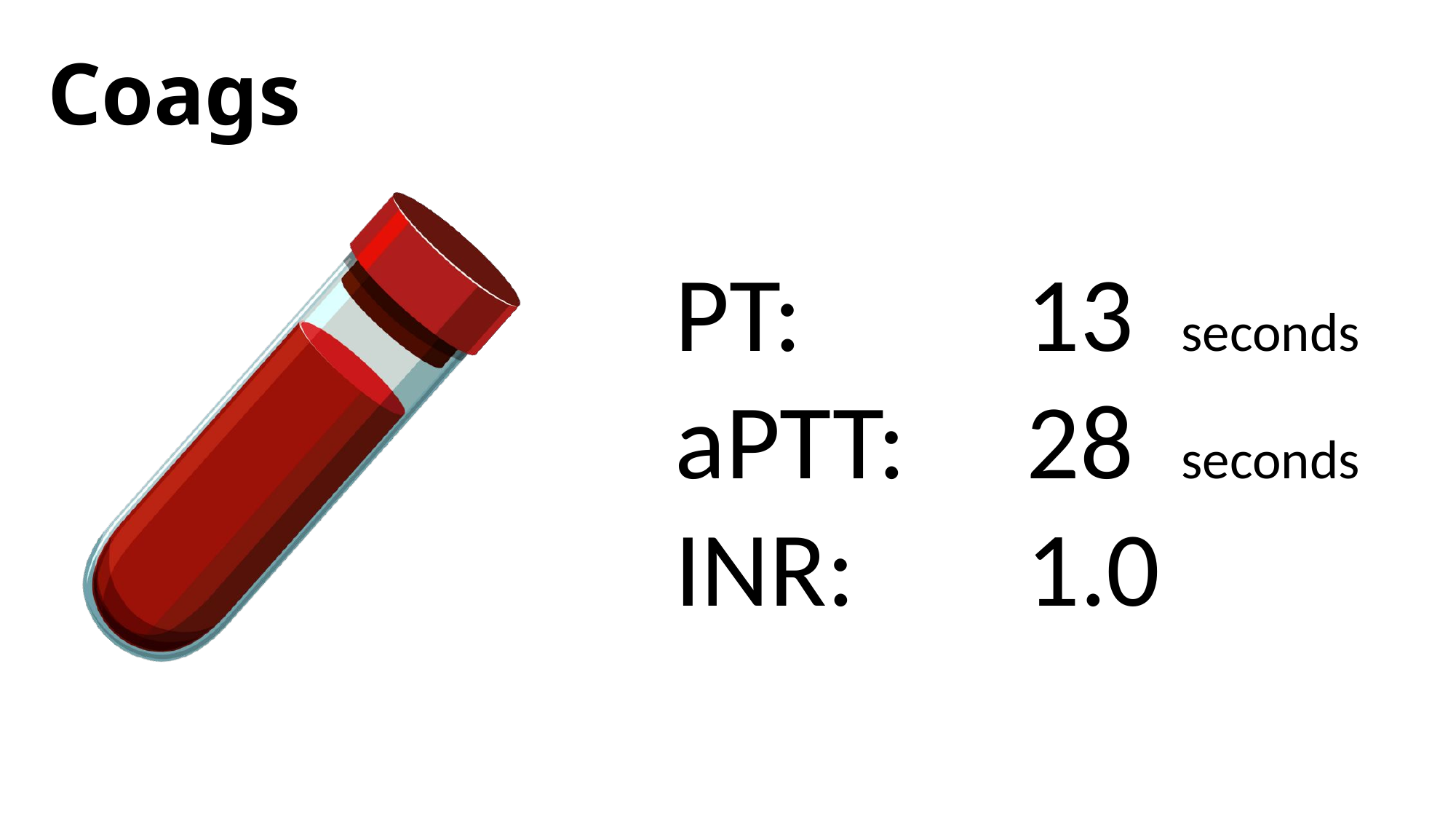

# Coags
PT:	 	 13 seconds
aPTT:	 28 seconds
INR:		 1.0

## Slide 8
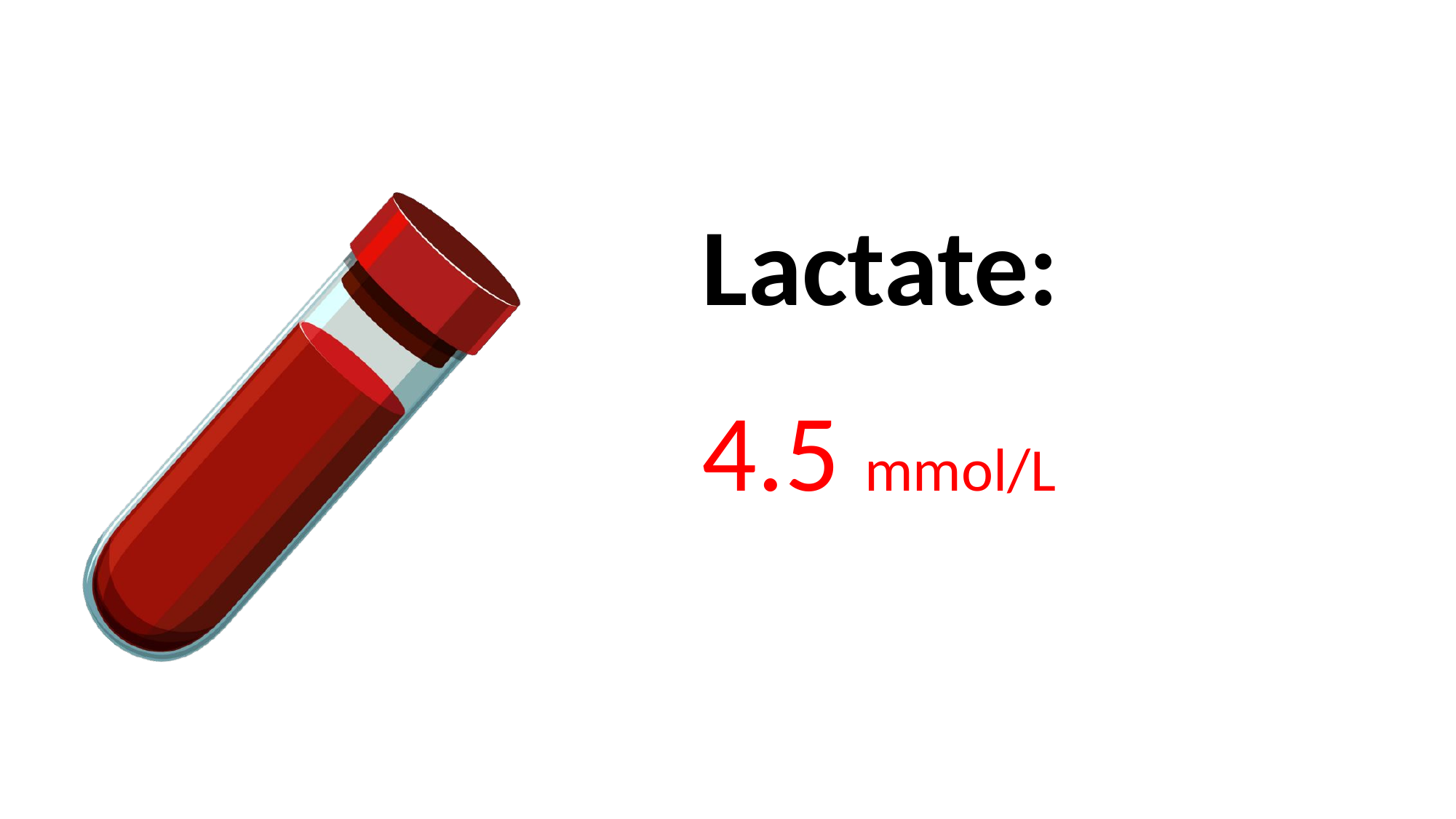

Lactate:
4.5 mmol/L

## Slide 9
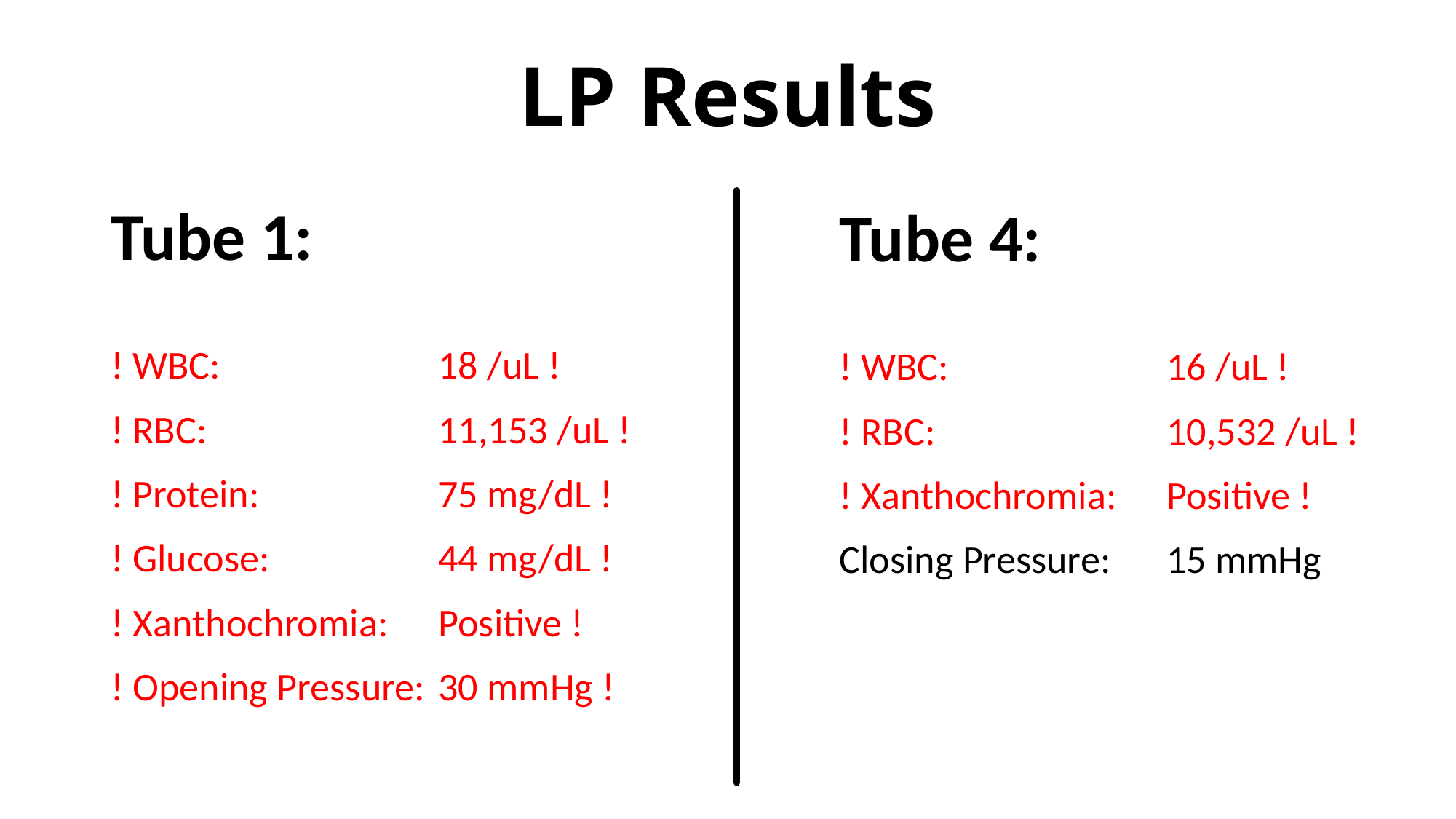

# LP Results
Tube 1:
! WBC:		18 /uL !
! RBC:			11,153 /uL !
! Protein: 		75 mg/dL !
! Glucose: 		44 mg/dL !
! Xanthochromia:	Positive !
! Opening Pressure:	30 mmHg !
Tube 4:
! WBC:		16 /uL !
! RBC:			10,532 /uL !
! Xanthochromia:	Positive !
Closing Pressure: 	15 mmHg

## Slide 10
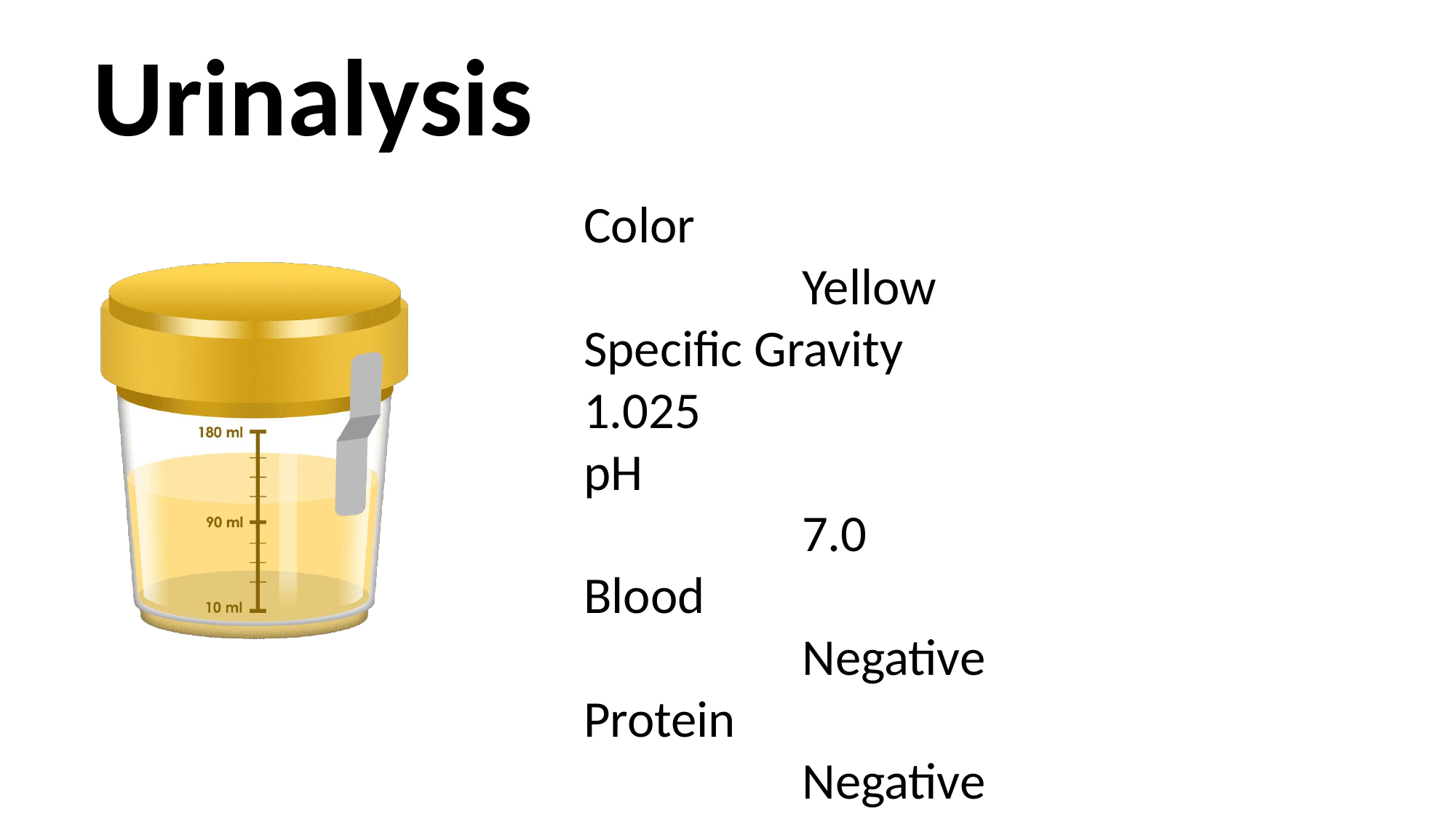

Urinalysis
Color								Yellow
Specific Gravity					1.025
pH									7.0
Blood								Negative
Protein								Negative
Glucose							Negative
Ketones							Negative
Leukocyte Esterase			Negative
Nitrates							Negative

## Slide 11
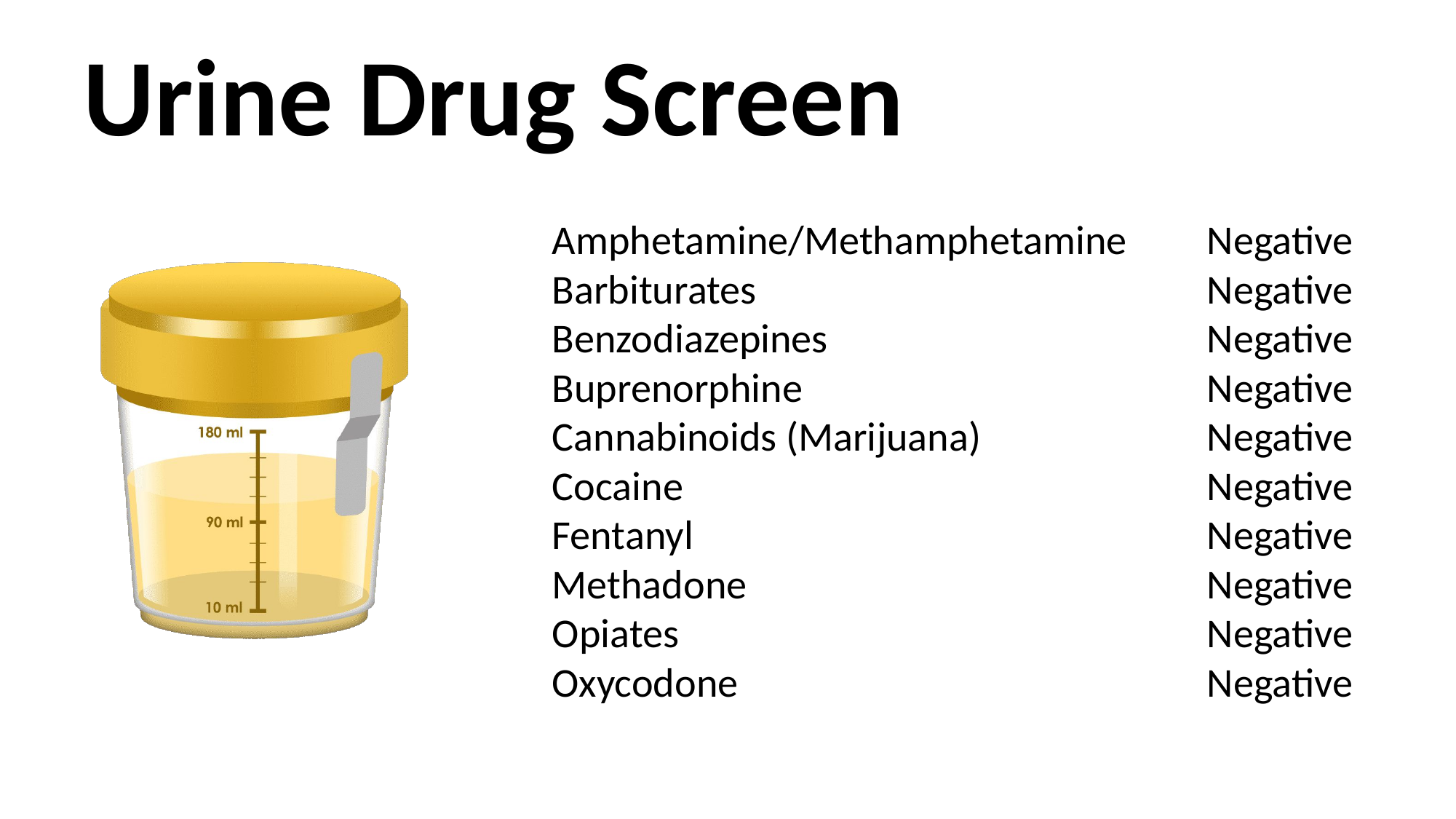

Urine Drug Screen
Amphetamine/Methamphetamine	Negative
Barbiturates					Negative
Benzodiazepines				Negative
Buprenorphine				Negative
Cannabinoids (Marijuana)			Negative
Cocaine					Negative
Fentanyl					Negative
Methadone					Negative
Opiates					Negative
Oxycodone					Negative

## Slide 12
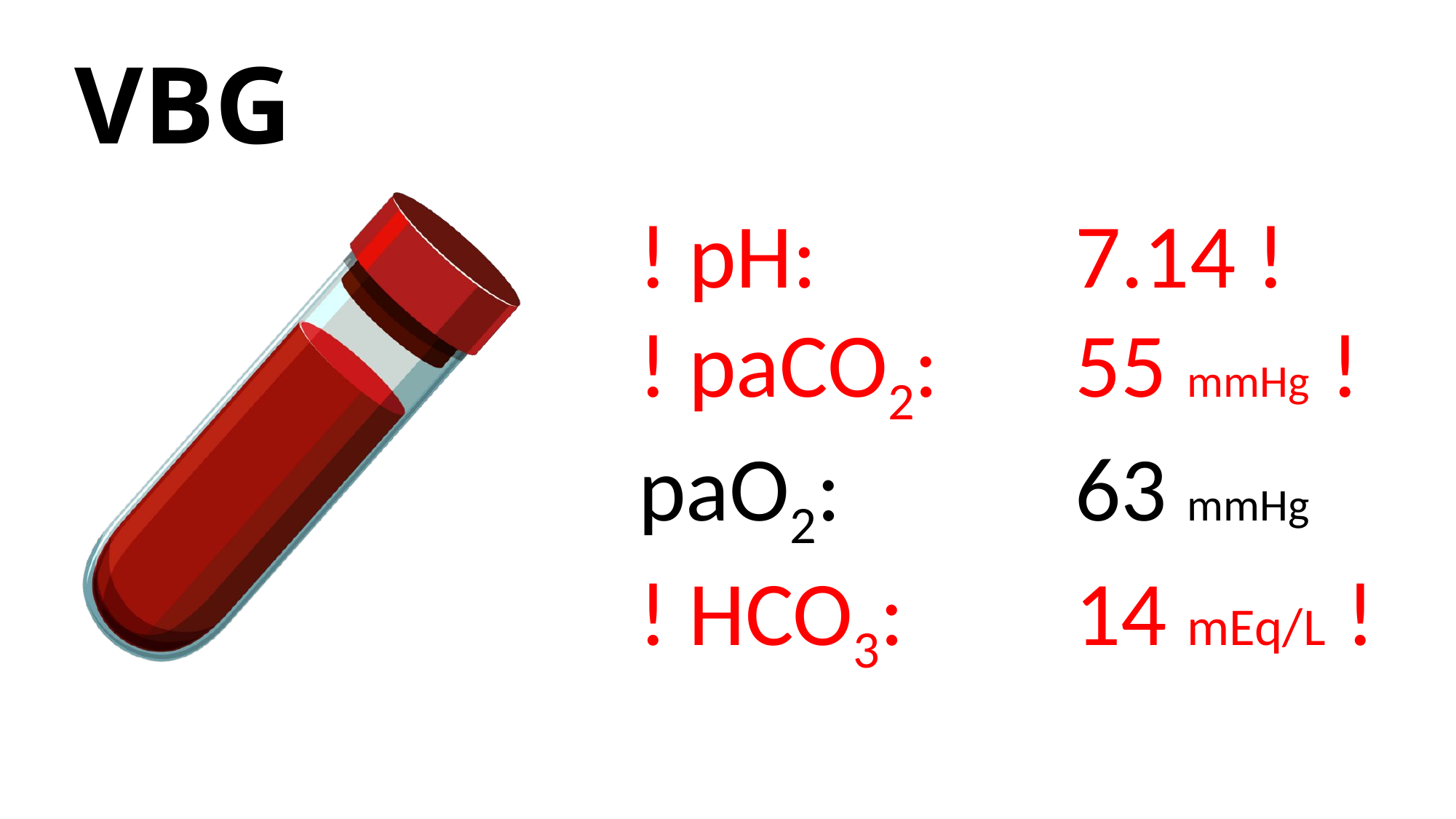

# VBG
! pH: 			7.14 !
! paCO2:		55 mmHg !
paO2:			63 mmHg
! HCO3:		14 mEq/L !

## Slide 13
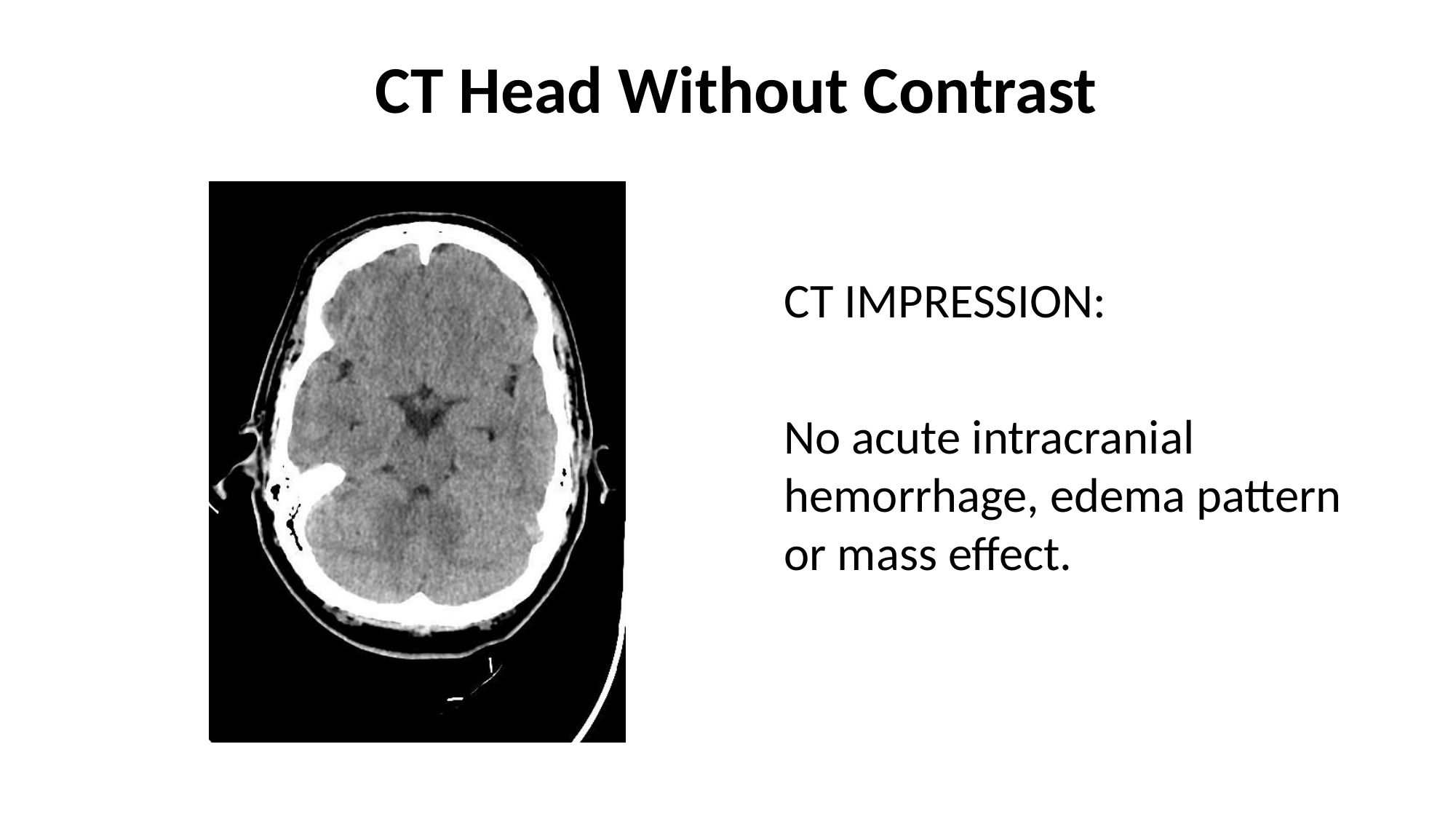

# CT Head Without Contrast
CT IMPRESSION:
No acute intracranial hemorrhage, edema pattern or mass effect.

## Slide 14
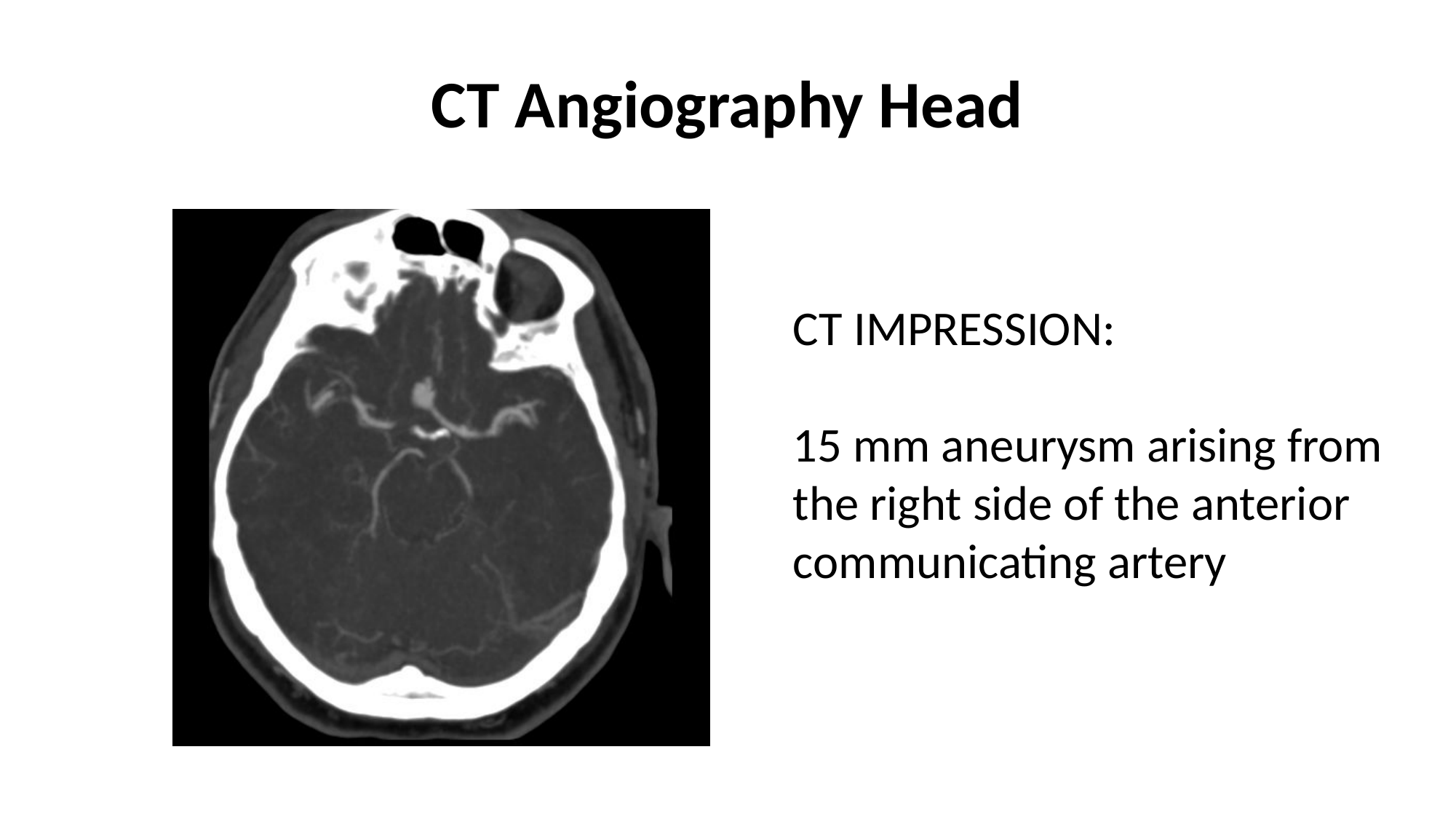

# CT Angiography Head
CT IMPRESSION:
15 mm aneurysm arising from the right side of the anterior communicating artery
